# Evaluating eukaryotic secreted protein prediction

**DOI:** 10.1186/1471-2105-6-256

**Published:** 2005-10-14

**Authors:** Eric W Klee, Lynda BM Ellis

**Affiliations:** 1 Department of Laboratory Medicine and Pathology, University of Minnesota, Mayo Mail Code 609, 420 SE Delaware Street, Minneapolis, MN 55455, USA

## Abstract

**Background:**

Improvements in protein sequence annotation and an increase in the number of annotated protein databases has fueled development of an increasing number of software tools to predict secreted proteins. Six software programs capable of high throughput and employing a wide range of prediction methods, SignalP 3.0, SignalP 2.0, TargetP 1.01, PrediSi, Phobius, and ProtComp 6.0, are evaluated.

**Results:**

Prediction accuracies were evaluated using 372 unbiased, eukaryotic, SwissProt protein sequences. TargetP, SignalP 3.0 maximum S-score and SignalP 3.0 D-score were the most accurate single scores (90–91% accurate). The combination of a positive TargetP prediction, SignalP 2.0 maximum Y-score, and SignalP 3.0 maximum S-score increased accuracy by six percent.

**Conclusion:**

Single predictive scores could be highly accurate, but almost all accuracies were slightly less than those reported by program authors. Predictive accuracy could be substantially improved by combining scores from multiple methods into a single composite prediction.

## Background

Predicting secreted proteins from primary sequence is a major component of automated protein annotation and is critical to a wide range of studies. Embryology, tumor maker detection, and agricultural animal performance are investigated using eukaryotic secreted proteins and their role in cell-to-cell communication, cellular differentiation, morphological development, and cellular response to disease. Many software tools have been developed for *ab initio *cellular localization prediction, using machine learning techniques such as neural networks, hidden Markov models and support vector machines. Identifying the program best suited for a researcher's needs requires familiarity with several different programs. Prediction accuracy depends on the methods employed by a program and the integrity of the data used to develop the program. Additionally, unbiased comparison using an independent protein sequence set is needed to compare programs, as system characteristics reported by program authors are often inflated [1].

The ambiguity of terminology used to describe and label secreted proteins often results in confusion on just what type of protein is being predicted or discussed. To eliminate this confusion, biologically concrete labels will be used in lieu of the term "secreted protein" or "secretory protein", here. Proteins possessing an N-terminal signal sequence and entering the classical secretory pathway via the endoplasmic reticulum, will be called CoTranslationally Translocated (CTT) proteins. Proteins transported out of the cell (regardless of mechanism) will be called extracellular proteins, proteins exported through the CTT pathway will be called classical extracellular proteins, and proteins exported by other mechanisms will be called non-classical extracellular proteins.

Most prediction programs predict CTT (not extracellular) proteins by identifying an N-terminal signal sequence, a signal sequence cleavage site, or a combination of both features, in a target sequence. New programs try to improve this approach through refinements in the sequence data used for program development and the application of new decision making algorithms to the problem. Novel methods, including predictions based on base composition across the entire protein sequence, identifying localization specific protein domains, homology to annotated protein databases, and mining partial protein annotations for key words, are also being used. Though programs that predict signal sequences often also predict signal sequence cleavage sites, we here focus on the former, since the latter has been recently reviewed [2]. We also focus on prediction of eukaryotic signals; the prokaryotic signal pathway has been recently reviewed [3].

Here, six programs, selected for their applicability for high throughput analysis, are described, and their ability to predict CTT proteins in eukaryotic proteins, are evaluated: SignalP 3.0 [4], SignalP 2.0 [5], TargetP 1.01 [6], PrediSi [7], Phobius [8], and ProtComp 6.0 [9]. SignalP 3.0, Phobius, PrediSi, and ProtComp 6.0 are recently released and have not been extensively reviewed nor independently compared. TargetP 1.01 and SignalP 2.0 are older programs, previously demonstrated to have high accuracy; they provide a basis for comparing our results with other studies [1,10-12].

### Prediction programs

SignalP versions 2.0 and 3.0 both use Neural Networks (NN) and Hidden Markov Models (HMM) to predict CTT proteins, through the analysis of protein sequence N-termini. These programs are among the most accurate methods for CTT protein prediction [1,10,11] and the programs' HMMs have an uncommon ability to discriminate N-terminal signal peptides from N-terminal signal anchors. SignalP 2.0 neural networks were trained using N-terminal subsequences containing CTT signal peptides and subsequent 30 residue of the mature peptides of 1137 eukaryotic CTT proteins and 70 residue N-terminal subsequences of 1451 eukaryotic non-CTT proteins, abstracted from SwissProt 35.0. SignalP 2.0 outputs four predictors computed by independent neural networks and two predictors computed by the Hidden Markov Models. NN outputs include the position and probability of the residue most likely to belong to a signal peptide (S-score max), the average probability all residue analyzed belong to a signal peptide (S-score mean), the position and probability of the residue most likely to be the first N-terminal residue of the mature peptide (C-score max), and a geometric average of the C-score and smoothed derivative of the S-score (Y-score). For each predictor a Boolean flag denoting CTT or non-CTT protein is returned, along with a composite neural network prediction which identifies CTT proteins in sequences which possess a high average S-score from the first N-terminal residue to the residue with the maximum Y-score, followed by a predicted cleavage site.

SignalP uses two HMM's, one that models the CTT signal peptide and a second that models a signal anchor. An N-terminal signal peptide is a short polypeptide (average length 20–25 residues), has no strongly conserved sequence motifs, but has three distinct sequential regions, the n (N-terminal)-region, the h (hydrophobic)-region, and the c (C-terminal)-region [13,14]. The signal peptide model contains submodels that describe each of these three regions. The signal anchor model contains two submodels that represent its n-region and h-region. In the signal peptide model, the h-region is limited to between six and twenty residues, the n-region must have at least one residue (and start with a methionine), and the c-region must have at least three residues. The n-region and c-region contain self-cyclic states with exponentially decaying transitions. This type of transition state allows the model to fit signal peptides possessing n-regions and c-regions with variable lengths, while still constraining the system, preventing unusually long region lengths, and thereby encapsulating the known properties of these regions. In the signal anchor module, the architecture of the n-region is the same, but the h-region also possesses a self-cyclic, exponentially decaying, transition state. The HMM outputs the position and score of the residue with the maximum C-score and a mean S-score for the entire sequence analyzed. In addition, Boolean flags for both predictors and a composite predictor characterizing the analyzed sequence as CTT, signal anchor, or other, are also output [5,15].

SignalP 3.0 possesses updated neural network architecture, new selection criteria for training sequences, and a composite score for signal peptide prediction. The neural networks were modified to include input nodes for sequence composition characteristics. Also, a symmetric sliding window of size 27 for signal peptide prediction and an asymmetric window of size 24 for cleavage site prediction were implemented after an exhaustive analysis of 27,000 neural networks determined these non-uniform window sizes provided the best performance. The networks were retrained using protein sequences from SwissProt 40.0, which were filtered to remove sequences likely to be mis-annotated. The new filtering process limited eukaryotic training data to sequences containing an alanine, cysteine, glycine, leucine, proline, glutamine, serine, or threonine at the first position upstream of the annotated cleavage site [4]. Additionally, ProP [16] predictions were used to identify and remove ten sequences likely to contain mis-annotated signal peptide cleavage sites. Finally, the D-score, computed from the mean S-score and the maximum Y-score, was added, thereby incorporating data from cleavage site predictions into the signal peptide predictions and improving their accuracy [4].

SignalP 2.0 and SignalP 3.0 were evaluated and compared by the program authors using five-fold cross-validation. Overall, version 3.0 outperformed version 2.0 in cleavage site predictions and signal peptide presence predictions. Internal testing showed SignalP 3.0 NN and HMM differentiated CTT proteins from non-CTT proteins with 98% and 94% accuracy, respectively, and SignalP 2.0 NN and HMM differentiated these proteins with 97% and 94% accuracy, respectively. Accuracy was assessed for the analysis of the first 70 N-terminal residues of target proteins and accuracy may decrease if more residues are analyzed.

TargetP differs from the SignalP software by predicting CTT (SP) and mitochondrial (mTP) or chloroplastic (cTP) proteins through the analysis of N-terminal sequence data. The program has a two-layer architecture; the first layer uses independently-trained networks to predict SP, mTP, or cTP localization, and the second layer integrates first layer outputs into a final prediction. Non-redundant, equal size, sequence sets from SwissProt release 36 (for plants) and release 37 (for non-plants) were used to train the networks. cTP cleavage site predictions are performed using the methods implemented in ChloroP [17], SP cleavage site predictions are performed using the methods implemented in SignalP [15], and mTP cleavage site predictions are made with a motif identifying matrix. Both the overall prediction and individual numeric scores from each network are output. TargetP also assigns a reliability class (RC) to each prediction based on the difference between the highest scoring prediction and the second highest scoring prediction. TargetP was tested using cross-validation and shown to correctly predict CTT localization with 92% accuracy, 92% specificity and 95% sensitivity, in non-plant sequences. When compared to PSORT [18,19], MitoProt [20,21], ChloroP and SignalP, TargetP CTT predictions in non-plants had a higher specificity than PSORT and a higher sensitivity than SignalP [6].

PrediSi predicts CTT proteins through the analysis of N-terminal sequence data by positional weighted matrices. Matrices were developed for the n-region, h-region, and c-region of the signal peptide using 2,783 eukaryotic, 557 Gram-negative, and 236 Gram-positive CTT proteins and 5,547 eukaryotic, 2,013 Gram-negative, and 1,077 Gram-positive control sequences (cytoplasmic and nuclear), obtained from SwissProt 42.9. The resulting amino acid frequency values were corrected to account for baseline proteome levels. PrediSi outputs a single numeric score, predicted cleavage site and Boolean flag denoting CTT signal peptide presence or absence. Self-consistency testing correctly identified 72.66% of eukaryotic CTT proteins and correctly excluded 98.31% of control proteins. PrediSi was outperformed by SignalP-NN and SignalP-HMM in eukaryotic and Gram-negative predictions, but displayed improved performance in Gram-positive predictions. PrediSi is designed for extremely fast analysis and is well suited for high throughput processing. These valuable characteristics were achieved at the cost of slightly reduced accuracy [7].

Phobius predicts CTT proteins using Hidden Markov Models to analyze full-length protein sequences. The program also predicts transmembrane domains and is designed to differentiate N-terminal transmembrane domains from CTT signal peptides. The HMMs were trained using 146 sequences from the TMHMM dataset [22], 140 sequences from TMPDB [23], 2 sequences from the Moller dataset [24], and 4 TM sequences from SWISS-PROT. These sequences were divided into TM-only and TM-and-SP sequence sets. Additionally, SP-only and not-TM-not-SP sequence sets were created using SWISS-PROT 41.0 proteins. Phobius outputs a Boolean flag denoting the presence or absence of a CTT signal peptide, the number of transmembrane domains predicted and a position labeled protein orientation schematic. In ten-fold cross-validation testing, Phobius correctly predicted 91.1% of TM-and-SP sequences, 63.6% of TM-only sequences, 96.1% of SP-only sequences and 98.2% of not-TM-not-SP sequences. In comparisons to other programs, Phobius out performed TMHMM, HMMTOP, TMHMM – SignalP combination, and HMMTOP-SignalP combination predicting TM-and-SP sequences, while being outperformed by HMMTOP and TMHMM predicting TM-only sequences. None of the software's options which allow users to constrain predictions based using known information about the presence of CTT signal peptides and TM domains or use a homology modeling component to perform BLAST comparisons against NCBI's nr database, were used for the testing described here [8].

ProtComp 6.0, from Softberry, Inc., predicts protein localization, including extracellular proteins, using a combination of neural networks and sequence homology. Sequences are assigned localization through homology to experimentally and theoretically annotated databases, neural network predictions and pentamer distribution comparisons to the homology databases. Softberry reports 86% correct prediction of extracellular proteins as tested with approximately 200 extracellular proteins. In this study, only ProtComp neural network predictions were evaluated [9].

## Results

### Individual predictions

CTT signal peptide predictive accuracies for individual predictive scores are shown in Table 1. Based on Matthew's Correlation Coefficient (MCC) [25,26], SignalP 3.0 D-score was the most accurate predictor, closely followed by the SignalP 3.0 maximum S-score and the TargetP prediction. The most sensitive predictors were the SignalP 2.0 neural network Mean S-score and Hidden Markov Model S-probability score. The maximum prediction specificity was obtained using the SignalP 2.0 HMM maximum C-score predictor.

**Table 1:** System performance measures. Performance was measured based on the program's ability to correctly discriminate CTT proteins from non-CTT proteins. MCC = Mathews' Correlation Coefficient [1].

**Program**	**TP**	**FP**	**TN**	**FN**	**Sensitivity**	**Specificity**	**MCC**
TargetP	55	8	307	2	96%	87%	90%
SignalP3 NN – Cmax	52	48	267	5	91%	52%	62%
SignalP3 NN – Ymax	55	9	306	2	96%	86%	89%
SignalP3 NN – Smax	56	9	306	1	98%	86%	90%
SignalP3 NN – Smean	55	17	298	2	96%	76%	83%
SignalP3 NN – D	55	7	308	2	96%	89%	91%
SignalP3 HMM Cmax	46	9	306	11	81%	84%	79%
SignalP3 HMM Sprob	56	17	298	1	98%	77%	84%
SignalP2 NN – Ymax	56	14	301	1	98%	80%	86%
SignalP2 NN – Cmax	54	28	287	3	95%	66%	75%
SignalP2 NN – Smean	57	13	302	0	100%	81%	88%
SignalP2 NN – Smax	56	21	294	1	98%	73%	81%
SignalP2 HMM Sprob	57	21	294	0	100%	73%	83%
SignalP2 HMM Cmax	36	3	312	21	63%	92%	73%
Phobius	55	13	302	2	96%	81%	86%
PrediSi	52	12	303	5	91%	81%	83%
ProtComp NN	46	32	283	11	81%	59%	62%

### Combined predictions

The combinatorial analysis examined 14,892 unique predictor combinations; for each combination, all program performance measures were calculated. A maximum MCC value of 97% was obtained in 58 different score combinations. The t-score for the highest MCC value associated with the combinatorial prediction method (0.97) is t_s _= 76.8, corresponding to a significance level well below 0.05%. The Fisher's Z-transformation testing the significance between the combinatorial correlation (0.97) and the best correlation arising from a single prediction score (0.91) returned a p ≤ 1.72 e-14. These results support the significance of the reported finding.

The minimum number of predictors needed to obtain the 0.97 MCC value was four and occurred in five different combinations. In each of these combinations, the TargetP prediction, the SignalP 2.0 maximum Y-score and the SignalP 3.0 maximum S-score were included. The fourth predictor was the SignalP 2.0 mean S-score, SignalP 2.0 Hidden Markov Model S-probability, the SignalP 3.0 mean S-score, the SignalP 3.0 D-score, or the SignalP 3.0 Hidden Markov Model S-probability. The most accurate pairs of predictors had MCC values of 95%: all included TargetP combined with either the SignalP 2.0 maximum Y-score, the SignalP 2.0 mean S-score, the SignalP 3.0 maximum S-score or the SignalP 3.0 D-score.

A prediction specificity of 98% was reached by 43 score combinations. The minimum number of scores required to reach this level of specificity was four and occurred in five different combinations. These five combinations all included the SignalP 2.0 maximum Y-score and SignalP 3.0 maximum C-score. The highest sensitivity obtained during the combinatorial analysis corresponded to the individual predictive scores with the highest sensitivity (combination set size 1), SignalP 2.0 NN mean S-score and SignalP 2.0 Hidden Markov Model S-probability.

Sequences can be analyzed using TargetP, SignalP 2.0 and SignalP 3.0 on the Vertebrate Secretome and CTT-ome Database [27]. Two sets of criteria can use used: positive TargetP prediction, SignalP 2.0 maximum Y-score and SignalP 3.0 maximum S-score (sensitivity 0.96, specificity 0.96, MCC 0.96), or positive prediction using TargetP or SignalP 3.0 D-Score (sensitivity 0.96, specificity 0.87, MCC 0.90).

## Discussion

The single most accurate predictors for discriminating CTT proteins from other proteins were the TargetP prediction, the SignalP 3.0 maximum S-score and the SignalP 3.0 D-score. The high accuracy of the SignalP 3.0 D-score is not a surprise, as it was designed to increase the overall prediction accuracy of CTT signal peptides, is itself a composite score of the neural network mean S-score and maximum Y-score, and incorporates cleavage site prediction information. Likewise, we expect the SignalP 3.0 maximum S-score to perform well, as this is obtained from networks trained on very current sequence data and the score is designed to specifically quantify CTT signal peptide presence. It is surprising the TargetP predictor performed so strongly, as it was expected predictors from older software, trained on older and generally smaller numbers of proteins, would be outperformed by the more recent programs. The TargetP accuracy can be attributed to its high specificity and ability to minimize false positive predictions; this is likely a result of TargetP's capacity to differentiate mitochondrial proteins from CTT proteins.

The CTT protein prediction sensitivity almost unilaterally decreased for common predictive scores shared by SignalP 2.0 and SignalP 3.0; the exceptions being the neural network maximum S-score and the Hidden Markov Model maximum C-score. Changes in specificity and accuracy were more variable, with the values of some predictors increasing and others decreasing for both performance measures. Exactly why the predictive sensitivity dropped between SignalP versions is not known, but it could be a by-product of the new screening protocols used to select positive CTT proteins for the version 3.0 training set. This protocol was particularly sensitive to the inclusion of protein sequences containing rare amino acids in the -1 and -3 residue locations relative to the CTT signal peptide cleavage site. While excluding these should improve the accuracy of cleavage site location prediction, it may have also caused the drop in CTT protein prediction sensitivity.

The PrediSi and Phobius predictions were 5% to 8% less accurate than the best predictors from TargetP and SignalP. While these programs fall short in predicting CTT proteins, they both possess characteristics that address niche analyses. The program developers state that the value of PrediSi is its computational speed. In our analysis this claim was validated; PrediSi was clearly the fastest program evaluated and did not restrict the size of sequence set analyzed. Therefore, if users are working with extremely large datasets, PrediSi can be used for rapid initial screens. However, for more accurate results, PrediSi's analyses should be combined with more rigorous, computationally expensive, methods.

The results obtained for Phobius are not surprising, as this program was not developed to specifically differentiate CTT proteins from non-CTT proteins, but to differentiate CTT signal peptides from N-terminal transmembrane domains. Since proteins with N-terminal transmembrane domains were intentionally not included in the test set, we could not assess this function. Phobius, like PrediSi, is not as accurate as TargetP and SignalP for CTT protein prediction. However, Phobius could add value to an analysis pipeline processing protein sequences containing N-terminal transmembrane domains.

ProtComp 6.0 returned the most disappointing results, despite the possibility of inflated results due to duplication between the test set and neural network training set. This program is not designed to strictly identify CTT proteins, but predicts localization to multiple cellular compartments. As such, quantifying CTT proteins required combining multiple prediction categories output by the program, which may have added to the poor performance. ProtComp is the only program tested which differentiates extracellular proteins from other CTT proteins. However, it is unclear if this program predicts non-classical extracellular proteins. ProtComp is more suitable for general localization screening than for specific locale predictions, as discussed here.

Combinatorial analysis is a systematic method for identifying the complementary CTT protein predictors best suited for incorporation into an analysis pipeline. A maximum combination of six scores was chosen to limit the exponential explosion of combinations evaluated, while still allowing for a single predictive score from each program to be included in the optimal combination. Fifty-eight different combinations provided a CTT signal peptide prediction accuracy of 97%, which exceeded the highest single score accuracy by 6%. A minimum of four predictive scores was necessary to obtain this accuracy and occurred in five different combinations. Interestingly, the TargetP prediction, the SignalP 2.0 maximum Y-score and the SignalP 3.0 maximum S-score, but not the SignalP 3.0 D-score (one of the highest individual scores), were included in all five. The fourth predictor in these five combinations varied. Accuracy was only slightly reduced (decreased by less than 1%) when the fourth predictor was eliminated.

It is possible the combinatorial predictors identified in this study may prove to be non-optimal in large applications, since the optimal 4-predictor combinations may have been over-fit to the data. The combination of the TargetP prediction, the SignalP 2.0 maximum Y-score and the SignalP 3.0 maximum S-score, the three predictors common to all five of the most accurate predictor combinations, may be best used in an analysis pipeline, to avoid the over-fitting while still generating high accuracy predictions. These are the three used for multiple predictor sequence analysis on the Vertebrate Secretome and CTT-ome Database [27].

It has been suggested that program authors overstate the predictive accuracy of their programs [1]. Almost all of the predictive accuracies reported here were lower than those reported in the original publications. TargetP was reported to correctly predict localization with 92% MCC accuracy, slightly higher than the 90% we calculated. For the SignalP 2.0 Hidden Markov Model the highest predictive score had an accuracy of 83% MCC, 11% lower than the published accuracy. The program's neural network accuracies, however, were comparable, with a published accuracy of 87% and an accuracy of 86% found in our testing. The published accuracy of the SignalP 3.0 neural network predictions was 7% higher and that of the Hidden Markov Model predictions was 10% higher than the accuracies obtained in our testing. Phobius predictions were reported to be 96.1%, 10% higher then the accuracy found in this study. PrediSi is one of the only programs to report a lower accuracy that what was found in this testing; reporting 73% accuracy compared to the 83% in this evaluation. ProtComp 6.0 website reports correctly predicting extracellular sequences 86% of the time, 14% higher than the accuracy found in our testing. It is important to independently assess predictive performance.

## Conclusion

This study of eukaryotic CTT protein prediction software evaluates six programs. Each offers a different analysis method, which in many cases is designed for a particular type of analysis. Understanding the differences between prediction programs is critical. The independent assessment of the predictive accuracy described here can provide a good basis for selecting software. TargetP, SignalP maximum S-score and the SignalP 3.0 D-score were shown to be the most accurate individual scores for CTT prediction. Prediction accuracy is significantly improved through use of multiple analysis methods and combining multiple predictive scores into a single composite prediction. Older predictive programs retain value; both SignalP 2.0 and TargetP contained predictive scores that were among the top predictive scores in both single and composite prediction analysis.

## Methods

### Protein test set

Full-length test proteins were abstracted from the SwissProt database. To eliminate bias caused by redundancy between the training and testing sequence sets only sequences modified or entered during 2004 (version 43.0+) were used for testing. *Homo sapiens*, *Mus musculus*, *Sus scrofa*, *Rattus norvegicus*, and *Bos taurus *proteins were identified using the SwissProt Sequence Retrieval System. For each organism the FASTA sequence and full SwissProt record were downloaded for proteins annotated as "Date=20040101:20041220 AND Comment Type=Subcellular Location". Sequences were categorized by the SwissProt record Subcellular Localization Comment entry and sequences annotated as "Putative", "Possible", "by Similarity", and, in one case, "by Similatity" were eliminated. Secreted, Mitochondrial, Nuclear, and Cytoplasmic proteins were selected from the remaining sequences and manually reviewed for ambiguous annotation. The final test set consisted of 372 full-length proteins; breakdown by cellular localization and organism is given in Table 2, the sequences themselves are in Additional file 1.

**Table 2:** Summary of the protein test set. 372 protein sequences from five vertebrate organisms and four localizations taken from the SwissProt database. The sequences themselves are in Additional file 1.

**Organism**	**Secreted**	**Mitochondrial**	**Nuclear**	**Cytoplasmic**	**Total**
Human	22	19	106	57	204
Mouse	19	10	37	35	101
Rat	7	2	14	21	44
Pig	4	0	3	4	11
Cow	5	3	1	3	12

**Total**	57	34	161	120	**372**

### CTT protein prediction

All CTT protein predictions were carried out using each program's web-servers. TargetP, SignalP 2.0 and SignalP 3.0 analyzed 125-residue, N-terminal subsequences of the 372 test proteins. TargetP was configured with non-plant settings, prediction of cleavage site enabled and default thresholds for assigning classifications. SignalP 3.0 was configured with settings for "Eukaryotic" sequence data, "Both" analysis methods, "No graphics" output, "Short" output and sequence truncation set to 70 residues. SignalP 2.0 was configured to analyze eukaryotic sequence data, output "no graphics", and otherwise use default parameters. PrediSi, Phobius and ProtComp analyzed the full-length test proteins. PrediSi was run using the eukaryotic organism group and a text based output. Phobius was run in normal prediction mode with short output. ProtComp predictions were executed on the animal and fungi ProtComp 6.0 server and positive CTT predictions assigned when the neural network predictions were extracellular, Golgi, endoplasmic reticulum or lysosome. Output files from all analyses were parsed, and program performance measures calculated, using custom Perl scripts.

### Program performance measures

To assess predictive accuracy, program sensitivity, program specificity (also known as positive predictive value), and program accuracy using MCC, were calculated. MCC value significance was validated using a standard t-test and Fisher's z-transformation [28]. For programs reporting multiple predictive scores, system characteristics were calculated for each score independently. Performance characteristics were also calculated for multiple combinations of predictors. An exhaustive analysis of two to six score combinations was performed. A protein was predicted to be secreted in a combinatorial analysis only when all predictive scores included in the combination independently predicted the protein to be secreted. The combinatorial analysis did not attempt to integrate or algorithmically combine the numeric scores values associated with each individual predictive score.

## Authors' contributions

EWK designed the evaluation, chose the six servers to be evaluated, designed the evaluation, analyzed the results, and drafted the paper; LBE supervised the overall work, and critically revised the paper. Both authors read and approved the final manuscript.

## Supplementary Material

Additional file 1 **Protein test set **372 eukaryotic Swiss-Prot protein sequences used to evaluate the six servers, in fasta format. Click here for file
